# Recycled Polypropylene Composites Reinforced with Microcellulose Fibres and Microcellulose-Derived Biochar: Thermal, Rheological and Mechanical Performance

**DOI:** 10.3390/ma19101942

**Published:** 2026-05-09

**Authors:** Wiktor Wyderkiewicz, Justyna Miedzianowska-Masłowska, Anna Sowińska-Baranowska, Marcin Masłowski

**Affiliations:** Institute of Polymer and Dye Technology, Lodz University of Technology, Stefanowskiego 16, 90-537 Lodz, Poland; wiktor.wyderkiewicz@dokt.p.lodz.pl (W.W.); justyna.miedzianowska@p.lodz.pl (J.M.-M.); anna.sowinska-baranowska@p.lodz.pl (A.S.-B.)

**Keywords:** recycled polypropylene composites, microcellulose fibres, microcellulose-derived biochar, upcycling of packaging waste, sustainable composites, circular economy

## Abstract

**Highlights:**

- Biochar retained most of the original microcellulose fibre morphology.
- MCF and BC nucleated rPP crystallization and increased stiffness.
- BC improved thermal stability and char residue of rPP composites.
- Low MCF loading increased flowability, while BC reduced melt flow indices.
- The approach supports circular use of PP waste and cellulosic biomass.

**Abstract:**

The mechanical recycling of mono-material biaxially oriented polypropylene (BOPP) packaging films produces recycled polypropylene (rPP) with degraded properties, limiting its use in higher-performance applications. This study investigates rPP reinforcement with 6–12 µm microcellulose fibres (MCFs, 2–10 pbw) and microcellulose-derived biochar (BC, 5–20 pbw), characterized by DSC, TGA/DTG, MVR/MFR, temperature-dependent rheology, mechanical testing and water contact angle (WCA) measurements. Both fillers acted as heterogeneous nucleating agents, shifting crystallization by up to 4 °C and increasing crystallinity by 2–4%. MCF introduced an additional low-temperature degradation step, whereas BC increased onset and peak degradation temperatures by up to 20 °C and increased char yield. Low MCF loadings increased MVR/MFR by 20–25% and reduced melt viscosity, while BC decreased flow indices by up to 50% and stiffened the melt. Tensile and flexural moduli increased by 15–25% with MCF and 40–50% with BC, with a stiffness–toughness trade-off at the highest BC contents. MCF reduced the water contact angle to 63.0° at 10 pbw, while BC increased it to 108.1° at 20 pbw, indicating opposite effects on surface wettability. Converting a single cellulosic feedstock into fibrous or carbonised fillers enables bio-based upgrading of rPP, in line with circular economy principles.

## 1. Introduction

The global production of plastic packaging has reached unprecedented levels, establishing polypropylene (PP) as one of the most widely utilized thermoplastics in the industry due to its favourable barrier properties, chemical resistance and mechanical performance [1,2]. Despite the theoretical recyclability of this material, the current waste management landscape presents a critical challenge. The European Union alone generates approximately 25.8 million tonnes of packaging waste annually, yet less than 30% of plastic packaging is currently recycled, with the remainder destined for incineration or landfill disposal [3,4]. This substantial stream of mono-material PP packaging films represents a significant resource that remains largely underutilized [5]. While mechanical recycling offers a viable pathway for material recovery and circular economy implementation, this approach is inherently limited by the inevitable degradation of the polymer matrix [6].

Addressing the issue of polymer downcycling requires a detailed understanding of the degradation mechanisms affecting polypropylene during mechanical recycling. Recycled PP typically exhibits a deterioration of mechanical and thermal properties arising from a combination of interconnected factors, including thermo-oxidative chain scission [7], molecular weight reduction, contamination originating from processing residues [8], and insufficient dispersion or interfacial interactions in composite systems [9]. Nasir et al. demonstrated that repeated thermo-mechanical stresses during extrusion and regranulation of recycled polypropylene promote thermo-oxidative degradation and chain scission, reflected in an increased melt flow index (MFI) and a loss of structural integrity [7]. These degradation processes lead to diminished tensile strength, reduced impact resistance, and compromised long-term performance [10]. Consequently, recyclates derived from PP packaging films are frequently downcycled into low-value applications rather than reintroduced into higher-performance material loops [8]. Improving the performance of recycled polypropylene composites has thus emerged as a critical research focus. To reverse this trend and engineer recycled PP materials suitable for demanding applications, the incorporation of reinforcing phases with proven compatibilization potential has been identified as a promising strategy.

Conventional approaches have traditionally relied on synthetic fibres (such as glass, carbon, and aramid fibres) and mineral fillers (including talc and calcium carbonate) to restore the stiffness and dimensional stability of recycled polypropylene, often at the expense of increased density and the introduction of additional non-renewable components into the material system [11]. However, this methodology presents a fundamental contradiction: introducing additional fossil-fuel-derived or inorganic components into the recycling stream undermines circular economy principles and perpetuates dependence on non-renewable resources [12]. Moreover, synthetic additives contribute an additional environmental burden through their manufacturing and processing, thereby increasing the overall carbon footprint of the material [13]. It is therefore increasingly recognized that a truly sustainable approach to recycled composite development must extend circular economy principles to encompass both the matrix material and the reinforcing phase.

One promising approach to upgrading recycled polypropylene involves the incorporation of biobased fillers, which not only enhance material properties but also contribute to sustainability by valorising biomass waste [2]. Bio-based fillers derived from renewable biomass resources offer a fundamentally different approach, one that is aligned with sustainability objectives and circular economy principles [12,14]. Among these candidates, cellulose-based reinforcements (including microcellulose fibres (MCFs), wood flour, and agricultural residues) have garnered considerable attention due to their renewability, high specific strength-to-weight ratios, and widespread availability [15]. Microcellulose fibres are characterized by a high aspect ratio and the inherent capacity to form rigid percolating networks within the polymer matrix, a structural arrangement that can significantly enhance the tensile stiffness and modulus of the recyclate [16,17,18]. Nevertheless, the practical utilization of cellulosic fillers in polypropylene matrices is often hampered by a fundamental thermodynamic incompatibility: the polarity mismatch between the hydrophilic cellulose surface and the hydrophobic polyolefin matrix [19]. This lack of affinity results in poor interfacial adhesion, moisture sensitivity, and a pronounced tendency toward agglomeration during thermo-mechanical processing, which can compromise the final mechanical performance [19,20]. To address these limitations, numerous studies have explored different compatibilization strategies aimed at improving the interfacial adhesion between hydrophilic cellulose and the non-polar polypropylene matrix. These approaches include the use of maleic anhydride grafted polypropylene (PP-g-MA), surface modification of cellulose (e.g., silanization or acetylation), as well as physical methods such as improved dispersion or processing optimization. Such approaches have been shown to enhance stress transfer, mechanical performance and the dispersion of cellulosic fillers in PP-based composites [19,20,21]. For instance, the use of MAPP as a compatibilizer in PP/nanocrystalline cellulose composites has been reported to significantly improve both mechanical and thermal performance through the formation of ester bonds at the cellulose–matrix interface [22]. In addition, recent studies have investigated the processing and rheological behaviour of PP/cellulose systems, demonstrating that filler morphology, surface chemistry, and interfacial interactions strongly influence melt viscosity, flow behaviour, and processability [16,17,23]. These effects have also been confirmed in recycled PP/cellulose composites, where oscillatory shear processing was shown to improve filler dispersion and reduce the activation energy of melt flow, even for rPP-based formulations [24]. These findings highlight that both structural and interfacial factors play a critical role in determining the final performance of PP/cellulose composites.

A novel strategy to overcome the inherent limitations of raw lignocellulosic fibres while retaining their ecological benefits is the thermal conversion of biomass precursors into biochar via pyrolysis [25]. This thermochemical transformation fundamentally alters the material characteristics of the organic precursor, converting a hydrophilic, moisture-sensitive fibre into a carbonised, carbon-rich solid with distinctly different surface properties [26,27]. Biochar not only acts as a stable reinforcing agent but also contributes to permanent carbon sequestration within the composite matrix, rendering the resulting materials potentially carbon-negative [28]. Unlike raw lignocellulosic fibres, biochar possesses a porous carbon structure with substantially reduced hydrophilicity, which markedly improves compatibility with the nonpolar PP matrix and enhances overall thermal stability [27,29]. Recent findings indicate that biochar can also function as a heterogeneous nucleating agent, favourably modifying the crystallization kinetics of polypropylene, specifically promoting the formation of crystalline structures that improve processing stability and final mechanical properties [29,30]. However, a notable gap persists in the literature regarding the systematic comparison of fibrous biofillers and their pyrolyzed derivatives within the specific context of recycled mono-material PP packaging films. In contrast to conventional approaches that treat biochar as an independent filler, this study adopts a unified strategy, in which both fibrous and carbonized fillers are derived from the same cellulosic precursor, enabling a direct comparison of morphology- and chemistry-driven effects on composite performance. The present study addresses this knowledge gap by developing and characterising sustainable composite systems based on recycled polypropylene (rPP) from mono-material BOPP packaging films, modified with microcellulose fibres (MCFs) and microcellulose-derived biochar (BC) as bio-based fillers. This integrated approach enables a systematic evaluation of how the transition from a fibrous, hydrophilic cellulosic structure to a carbonised, hydrophobic carbon skeleton influences the crystallization behaviour, thermal stability, melt processability and mechanical performance of the recyclate. By establishing clear structure–property relationships across a range of filler types and loading levels (2–10 pbw MCF and 5–20 pbw BC per 100 pbw rPP), this research aims to define the composition and processing windows necessary to produce functional recycled PP bio-composites with simultaneously improved thermal, rheological and mechanical performance, in line with circular economy principles.

## 2. Materials and Methods

### 2.1. Polypropylene Matrix

Biaxially oriented polypropylene (BOPP) post-industrial offcuts supplied by CDM Packaging (Ksawerów, Poland) served as the polymer matrix. The material was a transparent, unprinted packaging film originating from industrial processing of polypropylene. The molecular weight of the recycled polypropylene was not directly measured; however, melt flow rate (MFR) was used as an indirect indicator of molecular weight and degradation level.

### 2.2. Bio-Fillers

**Figure 1:**
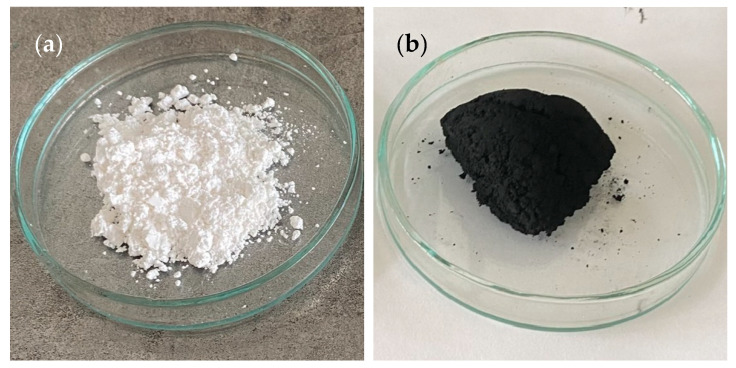
Arbocel UFC 100 cellulose microfibres (**a**) and biochar (**b**) obtained from Arbocel UFC 100 by pyrolysis.

Microcellulose fibres (MCFs) with an average fibre length of 6–12 µm (Arbocel UFC 100, RETTENMAIER Polska Sp. z o.o., Warsaw, Poland) were used as the primary bio-filler (Figure 1a). The material is a white, fibrous cellulose grade with a bulk density of 150–200 g·L^−1^ and a low ash content of 0.15 wt%. Before compounding, the cellulose fibres were dried in a laboratory convection oven (BINDER model FD-S 56, BINDER GmbH, Tuttlingen, Germany) to minimise residual moisture.

Biochar was produced from the same Arbocel UFC 100 cellulose by pyrolysis. The pre-dried fibres were heated to 600 °C for 2 h in a laboratory furnace (LIFT 3.0 BT, NEOTERM, Wrocław, Poland) under a nitrogen atmosphere, followed by cooling in a continuous nitrogen stream to prevent oxidation of the freshly formed biochar (Figure 1b). The carbon yield after pyrolysis was approximately 15 wt%, which is consistent with typical values reported for cellulose pyrolysis under similar conditions. The selected pyrolysis temperature of 600 °C represents a compromise between achieving sufficient carbonization and preserving the fibre-derived morphology of the precursor. This choice is further supported by our previous study, in which biochar obtained at 600 and 900 °C showed comparable yield and performance, while higher temperature did not provide significant advantages in morphology or reinforcing efficiency, despite a slight increase in carbon content [25].

### 2.3. Composition

The formulations of the polypropylene composites are summarised in Table 1. Recycled polypropylene (rPP) was used as the reference material, while Arbocel UFC 100 cellulose microfibres (MCFs) and the corresponding biochar (BC) derived from these fibres were incorporated as biofillers at different loadings. The contents of MCF and biochar were specified per 100 parts by weight (pbw) of rPP to enable a direct comparison between formulations.

The first series comprised rPP and rPP reinforced with 2, 5 and 10 pbw of cellulose microfibres, denoted as rPP MCF2, rPP MCF5 and rPP MCF10, respectively. In the second series, only biochar obtained from Arbocel UFC 100 was added to the matrix at 5, 10 and 20 pbw, giving the samples rPP BC5, rPP BC10 and rPP BC20.

The selected loadings of microcellulose fibres (2–10 pbw) and biochar (5–20 pbw) fall within the typical ranges reported for MC- and biochar-reinforced polypropylene composites, where 5–10 wt% is commonly used to balance stiffness enhancement and processability for cellulose fillers, and 5–20 wt% for biochar to optimize mechanical and thermal performance [27,31]. The upper loading limit for MCF was set at 10 pbw, consistent with literature reports indicating that higher cellulose fibre contents in polyolefin matrices tend to cause a pronounced increase in melt viscosity and a deterioration of filler dispersion homogeneity during melt processing, which compromises both processability and final specimen quality [31]. By contrast, the particulate morphology and reduced surface polarity of biochar are known to permit higher loading levels without the same dispersion limitations, justifying the extended range of 5–20 pbw for the BC series [27].

### 2.4. Preparation of rPP-Based Bio-Composite Compounds

In the first step, the post-industrial BOPP film waste was converted into recycled polypropylene granulate. The PP film was manually cut into smaller pieces and processed in a laboratory single-screw extruder (ZAMAK Mercator, Skawina, Poland) using a three-zone temperature profile of 180, 190 and 200 °C from the feeding section to the extrusion head. The continuous strand obtained at the nozzle exit was cooled on a conveyor belt equipped with a stabilising roller and then pelletised in a strand granulator (Brabender, Duisburg, Germany), yielding the reference rPP regranulate.

In the second step, this rPP granulate was used as the matrix for bio-composites. For each formulation listed in Table 1, rPP pellets were dry-blended with the required amount of microcellulose fibres (MCFs) or, in a separate series, with biochar (BC) obtained from MCF, and then melt-compounded again in the same ZAMAK Mercator extruder under identical temperature settings. The resulting composite strands were cooled and pelletised as before to obtain rPP MCF and rPP BC pre-compounds, which subsequently served as feedstock for injection moulding of test specimens.

A single-screw extruder was used to simulate processing conditions representative of typical mechanical recycling of polyolefin materials and to ensure consistent processing across all formulations. Although twin-screw extrusion is commonly used for industrial compounding of filled thermoplastics, a single-screw extruder was intentionally selected to reflect practical recycling conditions and to enable a consistent comparison of filler effects under non-optimized but industrially relevant processing scenarios.

### 2.5. Injection Moulding of rPP Bio-Composite Specimens

Test specimens for mechanical testing were produced via injection moulding using a Battenfeld PLUS 350 machine (KraussMaffei Group, Kottingbrunn, Austria). Granulates of rPP, rPP MCF and rPP BC pre-compounds were dried prior to processing and then fed separately into the injection unit for each formulation listed in Table 1. The barrel was operated with a two-zone temperature profile of 210 °C in the plasticising section and 220 °C at the nozzle, which lies within the recommended processing window for polypropylene-based materials.

**Figure 2:**
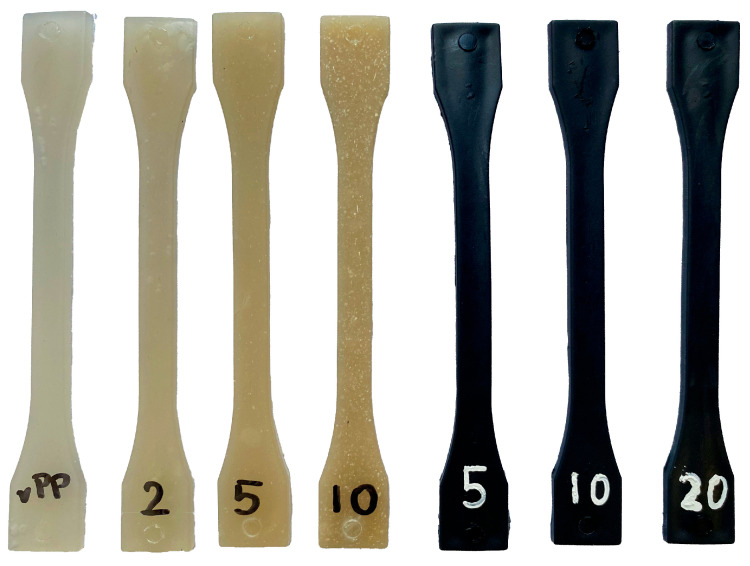
Injection-moulded dumbbell specimens of neat-recycled polypropylene (rPP) and rPP composites containing microcellulose fibres (MCFs, 2–10 pbw, left group) and microcellulose-derived biochar (BC, 5–20 pbw, right group).

Standard dumbbell and bar specimens (Figure 2) were produced for tensile, flexural, impact, hardness and thermal testing in accordance with the relevant ISO standards [32,33,34,35,36]. The moulded specimens were stored for at least 24 h after processing to allow internal stresses and structure relaxation to stabilise before testing.

### 2.6. Impact Strength

The impact resistance of the rPP bio-composites was evaluated using the Charpy method in accordance with PN-EN ISO 179-1 [32]. Tests were carried out on a Cometech QC-639P pendulum impact tester (Cometech, New Taipei City, Taiwan). For each bar specimen, thickness was measured at several positions with a digital caliper (0.01 mm resolution) and, in line with the standard, the smallest value (*h_min_*) was taken for subsequent calculations in order to avoid an overestimation of impact strength.

During the experiment, specimens were arranged flatwise on the supports of the pendulum rig and broken by a single strike of the Charpy hammer. Five specimens were tested for each material; the absorbed energy *E_s_* [J] shown by the instrument was recorded and then used to calculate the specific absorbed energy *E_sa_* [kJ/m^2^] according to

(1) $$E_{sa}=\frac{E_{s}}{b\times h_{min}}\times 10^{3}$$

where

*E_sa_*—the specific absorbed energy [kJ/m^2^];

*E_s_*—the energy absorbed during impact [J];

*b*—the specimen width at the impact plane [mm];

*h_min_*—the minimum measured specimen thickness [mm];

10^3^—the conversion factor for changing units from J/mm^2^ to kJ/m^2^.

### 2.7. Mechanical Properties Under Static Tensile Loading

The tensile response of the rPP bio-composites was evaluated under quasi-static loading in accordance with PN-EN ISO 527-1 [33]. Dumbbell specimens of type 1A obtained by injection moulding were tested on a universal testing machine, Zwick Roell 1435 (Ulm, Germany). Before testing, the actual thickness, *h*, and width, *b*, in the gauge section were measured at three random positions using a digital caliper with a resolution of 0.01 mm, and the lowest values were used for the calculations; each material variant was represented by at least three independent specimens.

The tests were carried out at a constant crosshead speed specified by the standard to ensure quasi-static deformation conditions. During loading, the machine continuously recorded force and displacement, which were converted into stress–strain curves and used to determine the tensile modulus (*E_mod_*), maximum tensile stress (*TS*), strain at maximum force (E_Fmax_) and elongation at break (E_b_).

### 2.8. Three-Point Bending

The flexural properties of the rPP bio-composites were determined in a three-point bending configuration according to PN-EN ISO 178 [34], using a ZwickRoell RetroLine universal testing machine (Ulm, Germany) equipped with a dedicated bending fixture. Before testing, the actual specimen width (*b*) and thickness (*h_min_*) were measured with a digital caliper (0.01 mm resolution), and the minimum recorded dimensions were used in the calculations; for each material, at least three bars were tested.

The specimens were simply supported on two parallel anvils at a fixed span and the load was applied at the mid-span by a central loading nose. A two-stage loading protocol was employed: after a 0.1 N preload, the crosshead speed was first set to 10 mm/min to accurately determine the flexural modulus and then increased to 50 mm/min to reach the maximum stress. The recorded force-deflection curves were processed to obtain the flexural modulus (*E_f_*), maximum flexural stress (*σ_fM_*) and corresponding strain (*ε_fM_*), which were later correlated with the tensile and impact results to assess the overall stiffness–toughness balance of the rPP, rPP MCF and rPP BC composites.

### 2.9. Hardness

The surface hardness of the rPP bio-composites was determined by Shore D indentation in accordance with PN-EN ISO 868 [35], using a Zwick 3105 durometer (Ulm, Germany) equipped with a conical indenter (30° tip angle, 0.1 mm tip radius) designed for harder thermoplastics and resins. Measurements were performed on standard injection-moulded bars; for each specimen, ten readings (five on each side) were taken with a dwell time of 5 s, and the arithmetic mean was reported as the Shore D hardness value.

### 2.10. Thermal Analysis (DSC/TGA)

The thermal transitions and stability of the rPP bio-composites were examined using a TGA/DSC 1 STAR System analyser (Mettler-Toledo, Greifensee, Switzerland). Approximately 10 mg of each composite was weighed into aluminium crucibles with perforated lids and conditioned in nitrogen prior to testing.

Thermogravimetric analysis (TGA) was first heated from 25 to 600 °C at 10 °C/min in an argon stream of 50 mL/min, which allowed the mass loss associated with decomposition of the polypropylene matrix and organic fraction of the fillers to be recorded. Afterwards, the purge gas was changed to air (50 mL/min) and heating was continued up to 900 °C so that the remaining char could be fully oxidised and the residual inorganic content could be determined.

Differential scanning calorimetry (DSC) measurements were performed on the same instrument using a three-step temperature programme at 10 °C/min: an initial heating from −50 to 200 °C, cooling back to −50 °C, and a second heating to 200 °C. The first heating removed the previous processing history, the cooling step provided crystallization temperatures and enthalpies, and the second heating yielded melting temperatures and enthalpies for rPP and the composites containing MCF or BC. The degree of crystallinity of the polypropylene phase was calculated from the DSC enthalpies using the relationship

(2) $$X_{c}=\frac{\Delta H}{w_{pp}\Delta H_{m}^{0}}\times 100\%$$

where ΔH is the absolute value of the melting enthalpy (or crystallization enthalpy) obtained from the DSC curves for a given sample, $w_{PP}$ is the mass fraction of polypropylene in the composite, and $\Delta H_{m}^{0}$ is the enthalpy of melting of a 100% crystalline polypropylene reference. In this work, a value of $\Delta H_{m}^{0}$ = 207 J/g was adopted according to literature data [37]. The same reference value was used for crystallinity calculated from both heating and cooling scans to allow direct comparison of neat rPP with the MCF- and BC-filled systems.

### 2.11. Melt Flow Index (MFI)

The melt flow behaviour of the recycled PP composites was determined using a Melt-Flow Plus tester (Karg Industrietechnik, Krailling, Germany) in accordance with PN-EN ISO 1133-1 [36]. Measurements were performed at 230 °C under a 2.16 kg load after a 240 s pre-heating period, using a capillary nozzle of 2.095 mm diameter and 8 mm length, with a piston travel of 25.4 mm.

For each formulation, the device simultaneously reported the melt mass-flow rate (MFR, g/10 min) and the corresponding melt volume-flow rate (MVR, cm^3^/10 min), calculated from the extrusion time of approximately 4 g of granulate over the set piston stroke.

### 2.12. Viscosity as a Function of Temperature

The rheological behaviour of the recycled PP composites was characterized on an ARES-G2 rotational rheometer (TA Instruments, New Castle, DE, USA) using 25 mm stainless-steel parallel plates. Test discs (≈2 mm thick) were pressed from granulate in a heated hydraulic press and conditioned for 24 h before measurements.

Each disc was clamped between the plates, preheated for 30 s at 130 °C, and then subjected to a linear heating ramp up to 280 °C at 5 °C/min under a constant 0.5 1/s shear rate. Throughout the run, the rheometer recorded the instantaneous temperature and the corresponding apparent viscosity, allowing assessment of how thermal softening and the presence of biofillers affect melt processability in the technologically relevant processing window.

### 2.13. Surface Wettability (WCA)

Static water contact angles were determined on injection-moulded bar specimens using a goniometer OCA 15 EC (DataPhysics Instruments, Filderstadt, Germany) to assess the surface wettability of neat rPP and the rPP-MCF/BC composites. A small droplet (3 µL) of deionised water was gently deposited onto the specimen surface using a microlitre syringe, and the droplet profile was recorded immediately after placement. For each image, the software fitted the liquid–solid interface and extracted the left- and right-hand contact angles; their arithmetic mean was taken as the local water contact angle. Ten droplets were analysed at different positions on each specimen, and the average value was reported as the characteristic WCA for a given formulation.

### 2.14. Optical Microscopy

The microstructure of the lignocellulosic MCF fibres and the derived BC particles was examined using an LAB40M optical microscope (Opta-Tech, Warsaw, Poland) equipped with Capture 3.0 analysis software. Dry powders were dispersed on glass slides to form a thin, single-layer coverage and observed in reflection mode without additional staining or etching, in order to preserve their native surface features. Images were acquired at magnifications of 50× and 100×.

### 2.15. Statistical Analysis and ANOVA

All measurements of WCA, MFI and mechanical properties, including impact strength, hardness, tensile strength, and three-point bending, were performed in replicates. The reported values represent the mean of the measurements and are accompanied by standard deviations to indicate the variability and reliability of the data.

To assess the significance of observed differences between composite formulations, a one-way analysis of variance (ANOVA) was conducted. The statistical analysis confirmed that variations in carbon filler type and additive content led to statistically significant differences in specific properties (*p* < 0.05).

## 3. Results and Discussion

### 3.1. Mechanical Properties

#### 3.1.1. Impact Strength

**Figure 3:**
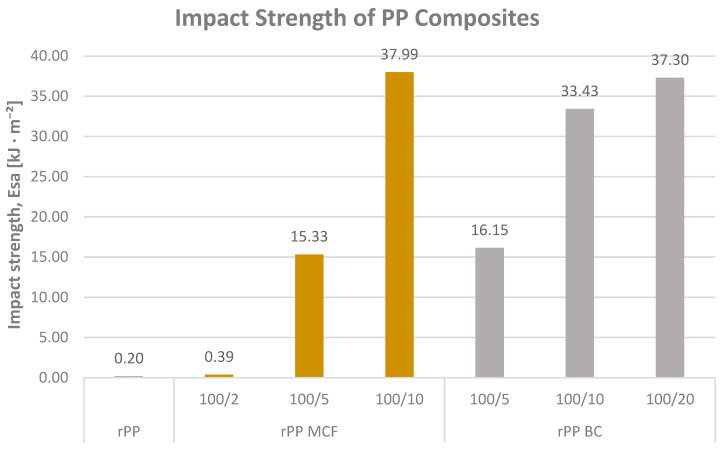
Impact strength (Esa) of recycled polypropylene (rPP) and rPP composites containing microcellulose fibres (MCFs) and microcellulose-derived biochar (BC).

The impact strength of neat rPP and of the composites filled with microcellulose (MCF) and microcellulose-derived biochar (BC) is shown in Figure 3. Neat recycled PP and the formulation with the lowest microcellulose content (rPP MCF2) exhibit very low nominal impact strength values of 0.20 and 0.39 kJ/m^2^, respectively. These values do not correspond to actual fracture energies but rather to the minimum energy recorded by the pendulum, because in both cases the specimens did not break during testing and therefore remained un-notched-bent instead of fully fractured.

Within the MCF series, increasing the fibre content to 5 and 10 pbw leads to a substantial rise in impact strength, reaching 15.33 and 37.99 kJ/m^2^, respectively. This pronounced improvement suggests the possible development of a reinforcing fibrous network that may contribute to crack resistance and impact energy dissipation; mechanisms such as fibre pull-out and matrix plastic deformation are consistent with the behaviour commonly reported for short-fibre thermoplastic composites [18], though further morphological analysis would be required for direct confirmation. A comparable trend appears for the BC-modified composites: rPP BC5, BC10 and BC20 reach 16.15, 33.43 and 37.30 kJ/m^2^, respectively, demonstrating that the carbonised, particulate phase derived from microcellulose can reproduce much of the toughening effect of the original fibres.

When both series are considered collectively, it becomes evident that above a critical bio-based filler content, recycled PP transitions from a non-fracturing behaviour to high and measurable impact energy absorption. In the case of MCFs, toughening may be associated with crack bridging and fibre pull-out mechanisms commonly reported for cellulosic fillers in polyolefin matrices [12], whereas BC may contribute through crack deflection and energy absorption at the filler–matrix interface; these interpretations remain consistent with the literature but would benefit from fracture surface characterisation for full confirmation [12,27]. Consequently, both modification strategies enable a comparable impact strength range.

#### 3.1.2. Tensile Strength and Elongation at Break

**Figure 4:**
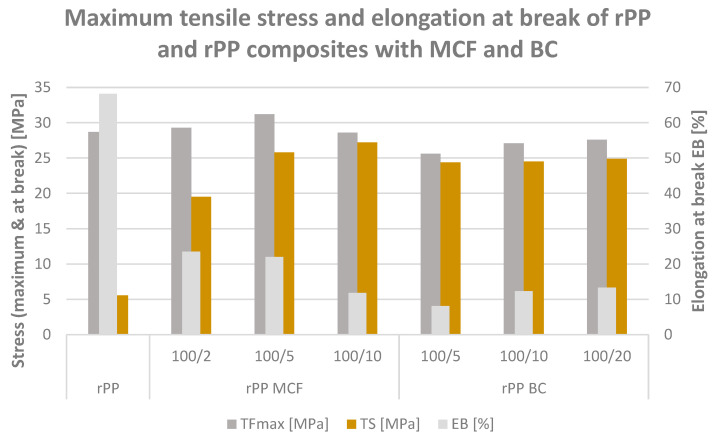
Maximum tensile stress (TFMax), stress at break (TS) and elongation at break (EB) of recycled polypropylene (rPP) and composites containing microcellulose fibres (MCFs) and microcellulose-derived biochar (BC).

**Figure 5:**
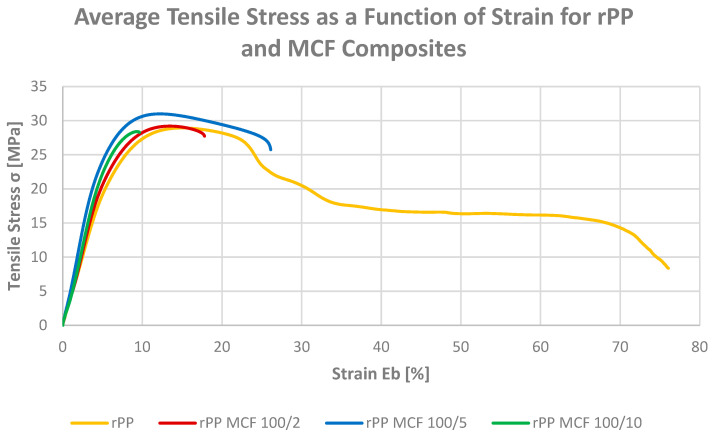
Stress–strain curves for recycled polypropylene (rPP) and composites containing microcellulose fibres (MCFs).

**Figure 6:**
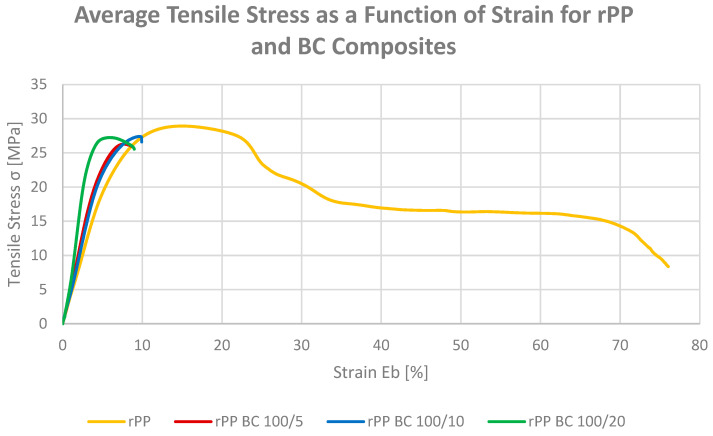
Stress–strain curves for recycled polypropylene (rPP) and composites containing microcellulose-derived biochar (BC).

The tensile testing was conducted to evaluate the effect of bio-based fillers on the strength and deformability of recycled polypropylene. The tensile strength and elongation at break of neat rPP and the rPP MCF and rPP BC composites are presented in Figure 4, Figure 5 and Figure 6. The incorporation of bio-based fillers considerably alters this balance between strength and ductility, with all composites showing higher tensile strength but reduced extensibility compared with neat rPP.

For the microcellulose-filled series, increasing the MCF loading from 2 to 10 pbw leads to a systematic rise in tensile strength from 19.5 to 27.2 MPa, while the elongation at break decreases from 23.5% to 11.8%. This behaviour is consistent with the expected load-bearing role of rigid, high-aspect-ratio fibres in constraining plastic deformation of the rPP matrix, which is also evident in the corresponding stress–strain curves (Figure 5) that display a steeper initial slope and a more pronounced maximum stress followed by limited strain hardening [37]. The data thus indicate that microcellulose effectively converts the very ductile recyclate into a stiffer and stronger material with a more engineering-like tensile response.

A similar, though slightly less pronounced, trend is observed for the composites containing microcellulose-derived biochar. At 5, 10 and 20 pbw BC, the tensile strength increases to 24.4–24.9 MPa, while the elongation at break is reduced to 8.1–13.3%, placing these materials in an intermediate regime between neat rPP and the highly reinforced MCF composites. The corresponding stress–strain curves (Figure 6) reveal a relatively sharp yield point and early onset of strain softening, consistent with the expected behaviour of rigid, particulate inclusions, which are generally reported to promote stress concentration and limit macroscopic drawability in polyolefin composites [27], while still providing a substantial gain in strength compared with the unfilled recyclate.

The difference in initial slope between the MCF and BC stress–strain curves at comparable elongation values reflects the distinct reinforcing mechanisms operative in each system. In the MCF series, stiffening arises from the load-bearing capacity of high-aspect-ratio fibrous particles capable of forming a partially percolating network within the rPP matrix and enabling efficient stress transfer [16,31]. In the BC series, the modulus increase is governed by the intrinsic rigidity of the carbonaceous particulate phase and its relatively higher compatibility with the non-polar rPP matrix [27,38], as evidenced by the elevated water contact angle and the pronounced melt viscosity increase observed in the rheological data. Regarding filler distribution, the optical micrographs in further studies confirm that both MCF and BC comprise in the micrometre range particles of comparable dimensions, with BC retaining the elongated, fibre-derived morphology of the parent cellulose after pyrolysis.

When both filler families are considered together, it becomes clear that fibrous MCF is more efficient than its biochar derivative in increasing tensile strength at comparable loadings, but this comes at the cost of a stronger reduction in elongation at break. In contrast, BC-filled systems offer a more moderate strength increase accompanied by slightly higher residual ductility, which may be advantageous for applications requiring a compromise between stiffness, strength and deformability in recycled PP-based formulations.

#### 3.1.3. Flexural Properties Under Three-Point Bending

**Figure 7:**
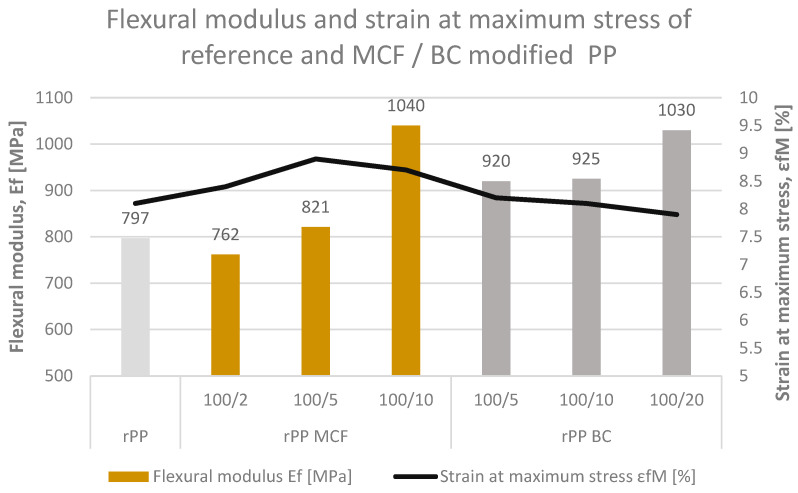
Flexural modulus, E_f_ (bars), and strain at maximum stress, ε_fM_ (black line), for neat recycled polypropylene (rPP) and rPP composites containing microcellulose fibres (MCF, yellow bars) or biochar (BC, grey bars). The sample notation 100/x denotes the rPP/filler weight ratio.

The three-point bending properties of the recycled PP composites are summarized in Figure 7 in terms of flexural modulus E_f_ and strain at maximum flexural stress ε_fM_. The reference rPP exhibits a flexural modulus of 797 MPa and ε_fM_ of 8.1%, reflecting a relatively soft but moderately deformable material. The incorporation of both microcellulose and microcellulose-derived biochar increases the flexural stiffness of the recyclate while only slightly affecting the strain at maximum stress.

For the MCF-modified series, the flexural modulus rises from 762 MPa at 2 pbw to 821 and 1040 MPa at 5 and 10 pbw, respectively, corresponding to an increase of up to about 30% relative to neat rPP. At the same time, ε_fM_ remains in a narrow range of 8.4–8.7%, indicating that the additional stiffness provided by the fibrous network does not lead to a pronounced loss of flexural deformability. This behaviour suggests efficient stress transfer from the rPP matrix to the high-aspect-ratio fibres under bending loads.

A comparable stiffening effect is observed for the biochar-filled composites. The flexural modulus increases to 920, 925 and 1030 MPa for rPP BC5, BC10 and BC20, respectively, placing these materials close to or slightly below the highly reinforced rPP MCF10 formulation. The strain at maximum stress is only marginally reduced to 7.9–8.2%, which indicates that the particulate BC phase can enhance flexural rigidity without severely compromising the ability of the specimens to sustain elastic-plastic deformation prior to failure. Overall, both MCF and BC effectively upgrade the flexural performance of recycled PP, with MCF providing slightly higher stiffness at a given loading, whereas BC offers comparable reinforcement in a more dimensionally stable, carbonaceous form.

From the perspective of designing injection-moulded parts from filled rPP, it is also important to note that both tensile modulus (E_mod_) and flexural modulus (E_f_) increase systematically with MCF and BC loading, as shown in Figure 4 and Figure 7. The resulting stiffness range covers approximately 15–25% enhancement for MCFs and 40–50% for BC relative to neat rPP, providing a practical modulus window for selecting suitable formulations for moulded components requiring defined rigidity levels.

#### 3.1.4. Hardness

**Figure 8:**
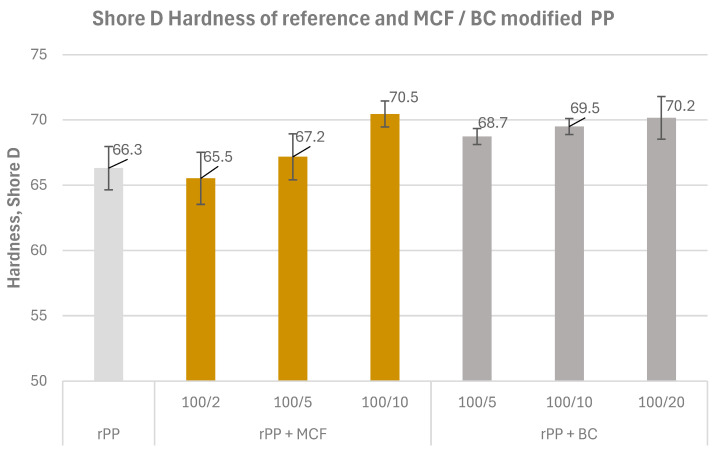
Shore D surface hardness of recycled polypropylene (rPP) and rPP composites filled with microcellulose fibres (MCFs) and microcellulose-derived biochar (BC).

Shore D hardness values for the rPP and for the MCF and BC composites are compiled in Figure 8. The average hardness of recycled PP was 66.3 ± 1.7 Shore D, whereas all filled systems exhibited higher hardness in the range of approximately 65.5–70.5 Shore D units. This indicates that the incorporation of bio-based fillers increases the surface stiffness of the recyclate and, at the same time, slightly reduces the scatter of the measurements, suggesting a more uniform macroscopic mechanical response.

The addition of microcellulose to the rPP matrix led to a gradual increase in hardness with increasing fibre loading. At the lowest content (rPP MCF2), the hardness remained close to that of the reference material, while for 5 and 10 pwb (rPP MCF5 and rPP MCF10) a clear rise to about 67–70 Shore D was observed, corresponding to an improvement of roughly several to more than ten percent compared with neat rPP. This trend confirms that the rigid fibrous MCF phase effectively stiffens the surface layer of the composite, although further increase in filler content may balance the reinforcing effect by reduced ductility and the possible formation of fibre agglomerates.

An even more pronounced hardening effect was obtained for the composites containing biochar obtained from microcellulose. For rPP BC5, rPP BC10 and rPP BC20, the average Shore D hardness increased systematically from 68.7 to 70.2, with the highest value recorded at the maximum biochar loading and accompanied by relatively low standard deviations. These results suggest that the transformation of hydrophilic microcellulose into a carbonaceous, biochar structure promotes the formation of a stiffer surface that is more resistant to local indentation, which is consistent with literature reports describing biochar as a rigid, well-compatible filler for polyolefin matrices [27,38].

### 3.2. Thermal Properties

#### 3.2.1. Differential Scanning Calorimetry (DSC) Analysis

**Figure 9:**
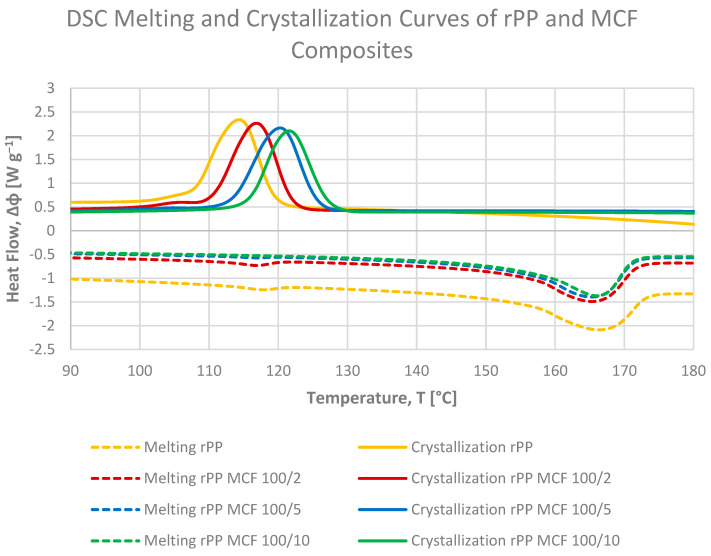
Heat-flow curves from DSC cooling and second-heating scans for recycled polypropylene and rPP composites with microcellulose fibres (MCFs), illustrating the effect of fibre loading on crystallization and melting behaviour; the upper set of curves corresponds to cooling, while the lower set represents the second heating run.

**Figure 10:**
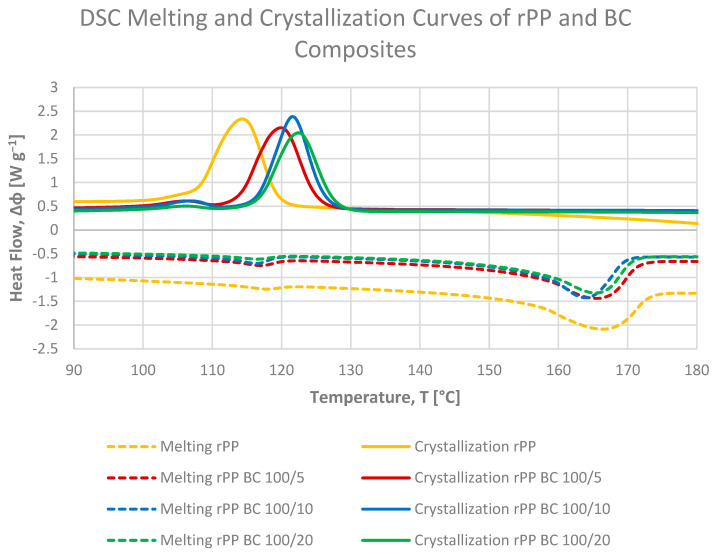
Heat-flow curves from DSC cooling and second-heating scans for recycled polypropylene and rPP composites with microcellulose-derived biochar (BC), illustrating the effect of BC loading on crystallization and melting behaviour; the upper set of curves corresponds to cooling, while the lower set represents the second heating run.

Differential scanning calorimetry was employed to evaluate the influence of microcellulose fibres (MCFs) and microcellulose-derived biochar (BC) on the melting and crystallization behaviour of recycled polypropylene (rPP). The DSC curves of all composites were shown in Figure 9 and Figure 10 and results summarized in Table 2 and Table 3.

The DSC thermograms display a single melting endotherm and a single crystallization exotherm (Figure 9 and Figure 10), which is typical of α-isotactic polypropylene, suggesting that, within the investigated composition range and processing conditions, the addition of both fillers does not lead to detectable polymorphic transitions or the formation of new crystalline phases [39]. The melting temperatures (Tm_peak_) remain within a narrow range of 163–166 °C for all samples (Table 3), confirming that the order and stability of the PP crystalline phase are only weakly affected by the presence of MCF or BC.

The degree of crystallinity calculated from the crystallization enthalpy during cooling (Xc from ΔHc, Table 2) remains within a relatively narrow range of approximately 40–43% for all compositions. Neat rPP exhibits Xc = 42.8%, while MCF- and BC-filled composites show values between 40 and 42%, indicating that the fillers mainly accelerate nucleation without substantially increasing the final crystalline fraction formed during cooling at the applied rate. The slight decrease in Xc for some MCF-containing samples may be related to restricted chain mobility in the vicinity of the fibre surface, which can limit lamellar thickening at later stages of crystallization [21].

The crystallization curves reveal a pronounced nucleating effect of both fillers. The crystallization onset temperature of neat rPP is approximately of 120 °C, whereas the addition of MCF shifts this value up to 127 °C at 10 pbw loading. A similar increase is observed for BC-filled systems, with Tc_onset_ reaching 128 °C for the formulation containing 20 pbw of BC. Similarly, the crystallization peak temperature (Tc_peak_) increases from 115 °C for neat rPP to 122 °C for MCF (10 pbw) and up to 123 °C for BC (20 pbw). These shifts demonstrate that both fibrous and carbonaceous particles provide efficient heterogeneous nuclei, allowing crystallization to start at higher temperatures during cooling and thus narrowing the processing window of the recyclate.

The crystallinity values determined from the second heating scan are systematically lower (about 31–37%), reflecting partial reorganization and melting-recrystallization phenomena upon reheating. For neat rPP, Xc from Hm is approximately 31%, and it increases to around 36% for rPP MCF5 and rPP BC20, demonstrating that both fillers slightly enhance the amount of stable crystals that survive the imposed thermal history. At comparable loadings, MCF tends to provide a somewhat higher crystallinity than BC at 5 pbw, whereas at higher filler contents (10–20 pbw) the differences between MCF and BC diminish, and both systems reach similar Xc values close to 36%. This behaviour suggests that, beyond a certain concentration, the overall nucleation density becomes sufficiently high for both fillers and further changes in filler morphology have only a minor impact on the crystalline fraction of the PP matrix.

Between the two filler types, MCF generally exerts a slightly stronger effect on shifting Tc to higher temperatures and at the same time stronger nucleating effect, consistent with its high surface area and good wetting by the PP melt, which facilitate efficient heterogeneous nucleation. BC, in turn, yields crystallinity levels comparable to MCF while offering a more thermally stable carbonaceous phase, which may be advantageous for applications requiring improved dimensional stability at elevated temperatures. Taken together, the DSC results confirm that both microcellulose fibres and their carbonised counterpart act as effective nucleating agents for recycled PP, subtly increasing the degree of crystallinity and strongly promoting earlier onset of crystallization, which correlates well with the enhanced stiffness observed in mechanical testing.

Moreover, the crystallinity trends extracted from DSC are broadly compatible with the mechanical performance of the materials. Composites with slightly higher Xc, such as rPP MCF5 and rPP BC20, also show an increased flexural modulus and tensile stiffness compared with neat rPP, indicating that the nucleation-induced enhancement of the crystalline fraction can contribute to stiffening of the recycled PP matrix. At the same time, the overall variation in Xc remains relatively small (on the order of a few percentage points), suggesting that the pronounced increases in stiffness are governed primarily by the load-bearing contribution of the rigid MCF and BC phases and by constraints imposed on the amorphous regions near the filler–matrix interface, rather than by bulk crystallinity alone.

#### 3.2.2. Thermogravimetric Analysis

**Figure 11:**
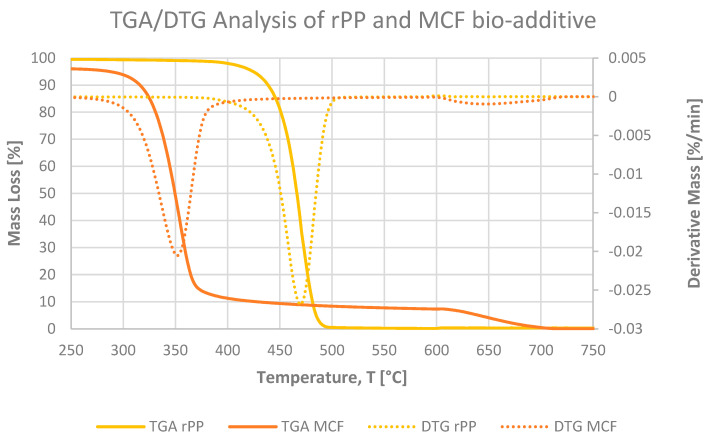
TGA and DTG curves of neat recycled polypropylene (rPP) and microcellulose fibres (MCFs) measured under the two-step programme, illustrating the much earlier onset and broader degradation of MCF compared with rPP and the formation of a larger char fraction in the cellulose additive.

**Figure 12:**
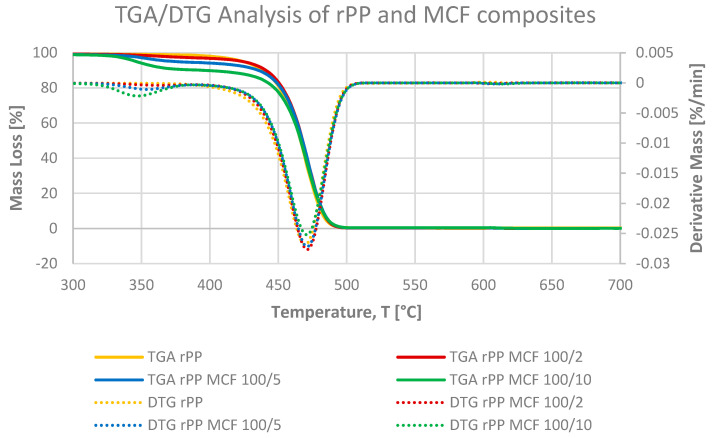
TGA and DTG curves of neat rPP and rPP-MCF composites, showing that increasing MCF loading shifts the initial mass loss towards lower temperatures, consistent with embedded fibre degradation, while the main DTG peak associated with PP chain scission remains at essentially the same temperature for all formulations.

**Figure 13:**
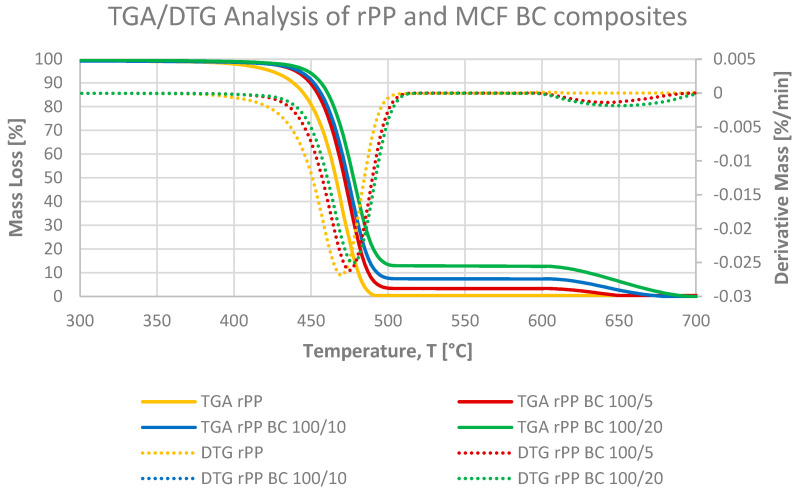
TGA and DTG curves of neat rPP and rPP-BC composites, demonstrating a systematic shift of both the onset and maximum of the main degradation step to higher temperatures and an increased residual mass at 700 °C with rising BC content, evidencing the stabilising and char-forming effect of the microcellulose-derived biochar phase.

Thermogravimetric analysis was used to assess the thermal stability of neat rPP, the corresponding MCFs and BC-reinforced composites and the standalone microcellulose additive under the two-step heating programme described in Section 2.10. The TGA and DTG curves of the rPP-based materials (Figure 11, Figure 12 and Figure 13) reveal a single dominant degradation step in the inert regime between approximately 380 and 500 °C, followed in the oxidative stage by the gradual mass loss associated with the burnout of the residual char and inorganic fraction [12].

In argon, neat rPP remains thermally stable up to roughly 350–370 °C, with a T_5_ of 425 °C and a DTG maximum at 469 °C and undergoes nearly complete thermal decomposition between 25 and 600 °C (ΔM_25–600_ = 99.9%). The absence of residue after thermal decomposition is typical of polyolefins. Neat microcellulose, by contrast (Figure 11), shows a small initial mass loss below about 150 °C, which stays well below 5% and can be attributed to the desorption of physically bound moisture inherent to the hygroscopic cellulosic structure. Upon further heating, MCF starts to decompose much earlier (T_5_ = 288 °C) and shows a broad DTG maximum at 352 °C with ΔM_25–600_ = 92.7%, which is typical for lignocellulosic materials undergoing extensive dehydration and char formation. In the rPP MCF composites (Figure 12), the degradation behaviour combines these two contributions: at 2 pbw MCF, T_5_ remains identical to neat rPP (425 °C), but at 5 and 10 pbw it shifts down to 375 and 347 °C, respectively, while T_DTG_ stays constant at 471 °C and ΔM_25–600_ remains above 99.6% for all MCF loadings. These results indicate that MCF reduces the thermal stability of the composite by lowering T_5_, initiating earlier mass loss, whereas the onset of fibre degradation, whereas the temperature of the main DTG peak is still dictated by bulk PP chain scission.

The microcellulose-derived biochar modifies this picture in a different way (Figure 13). In the BC-filled series, both the onset and the maximum of the main degradation step shift to higher temperatures: T_5_ increases to 441 and 443 °C for rPP BC5 and rPP BC10, and further to 449 °C at 20 pbw BC, while T_DTG_ moves from 475 °C at 5 pbw to 477 °C at 10–20 pbw. At the same time, ΔM_25–600_ decreases from 96.0% for 5 pbw BC to 92.38% and 87.22% at 10 and 20 pbw, respectively, reflecting the progressive buildup of a nonvolatile carbonaceous fraction already in the inert stage. This combination of higher T_5_, elevated T_DTG_ and reduced mass loss demonstrates that BC does not merely dilute the polymer phase but acts as a thermally robust, diffusion-limiting domain that slows down heat and mass transport through the composite, in agreement with previous reports on PP/biochar systems [39].

In the oxidative stage above about 600 °C, the TGA curves reveal a further decrease in mass that reflects the combustion of the char formed in the inert regime. For neat rPP and the rPP MCF composites, the mass has already fallen to almost zero by around 500–550 °C, and only a very shallow step remains up to 600 °C, which can be attributed to the final oxidation of minor carbonaceous residues originating from partially charred cellulose. In contrast, the rPP BC composites retain a clearly higher mass fraction at the end of the run, reflecting the high carbon content of the biochar phase. The residual mass increases systematically with BC loading: ΔM_25–600_ decreases from 96.0% at 100/5 to 92.4% at 100/10 and 87.2% at 100/20, while T_5_ rises progressively from 441 °C to 443 °C and 449 °C, and T_DTG_ shifts from 475 °C to 477 °C for both higher loadings. This demonstrates that the microcellulose-derived biochar contributes a thermally persistent carbon/inorganic skeleton that survives the main degradation of the polymer and remains only partially combusted within the investigated temperature range [39,40].

Overall, the combined information from Table 4 and Figure 11, Figure 12 and Figure 13 shows that microcellulose primarily introduces an additional low-temperature degradation contribution associated with fibre breakdown, while its carbonised derivative simultaneously increases char yield and elevates both the onset and peak temperatures of mass loss, thereby providing a tangible improvement in the high-temperature stability of the recycled PP composites. These thermal characteristics are consistent with the structural role of each filler: the less thermally stable, cellulosic MCF phase limits char formation and offers only a modest enhancement of high-temperature performance, whereas the thermally robust BC network, as confirmed by higher T_5_ and T_peak_ values together with markedly increased residual mass, corresponds to the greater stiffness, enhanced melt viscosity and increased hydrophobicity observed for the BC-filled formulations, thereby linking the TGA behaviour directly to the mechanical and rheological response of the materials.

### 3.3. Melt Flow and Temperature-Dependent Rheological Properties

#### 3.3.1. Melt Flow Indices (MVR/MFR)

**Figure 14:**
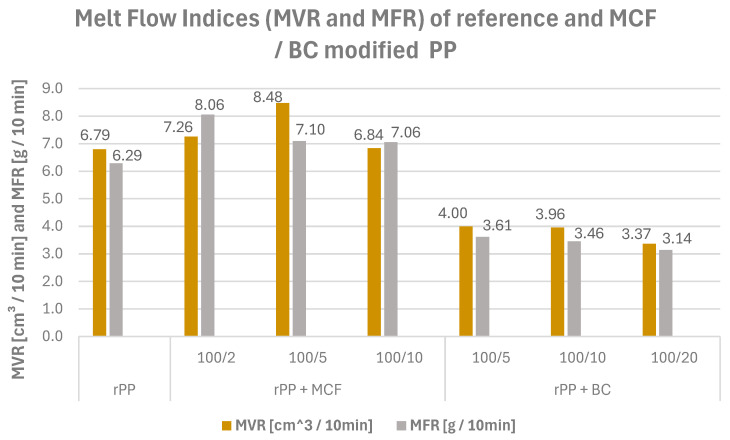
MVR and MFR of neat rPP and rPP-MCF/BC composites (230 °C/2.16 kg). MCF addition at low loadings slightly increases melt flow, while BC progressively reduces it, indicating a more pronounced viscosity-increasing effect of the carbonaceous filler.

The measured melt flow values (Figure 14) reveal how the addition of MCF and BC alters the processing behaviour of the recycled polypropylene matrix. Neat rPP exhibits an MVR of 6.79 cm^3^/10 min and an MFR of 6.29 g/10 min, placing it in the range of medium-flow polypropylene grades typically used for standard extrusion and injection-moulding operations [41].

Microcellulose has a rather moderate effect on these parameters. At 2 and 5 pbw MCF, the melt flow increases (MVR 7.25 and 8.48 cm^3^/10 min), whereas at 10 pbw, it returns close to the value of neat rPP (MVR 6.84 cm^3^/10 min; MFR 7.06 g/10 min). This behaviour suggests that low fibre loadings do not yet form a pronounced flow-hindering network, so the melt remains easy-flowing, while at higher loading, the developing fibre structure starts to counteract this effect by increasing resistance to flow in the molten state.

The behaviour of the BC-filled series contrasts sharply with that of MCF. For rPP BC5, BC10 and BC20, MVR drops to 4.00, 3.96 and 3.37 cm^3^/10 min, with corresponding MFR values of 3.61, 3.46 and 3.14 g/10 min, respectively. Thus, increasing BC loading systematically reduces melt flow, indicating a marked increase in apparent melt viscosity. This strong thickening effect is attributed to the rigid, high-surface-area biochar particles forming a dense, interacting network that restricts chain mobility in the melt, consistent with previous studies on PP/biochar and related biochar-reinforced thermoplastics [42].

#### 3.3.2. Temperature-Dependent Melt Viscosity

The apparent melt viscosity of neat rPP and the composites was measured during a linear heating ramp from 130 to 280 °C using an ARES-G2 rheometer in parallel-plate mode at a constant shear rate, i.e., at conditions comparable to the MFI temperature but under controlled, low-shear deformation. For the discussion below, attention is focused on the fully molten region around 230 °C, which is most relevant for extrusion and injection moulding. For neat rPP, the viscosity at 230 °C was 817 Pa·s.

**Figure 15:**
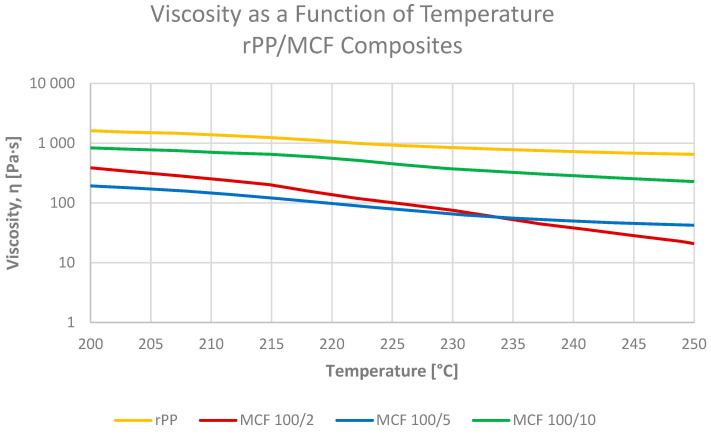
Apparent melt viscosity as a function of temperature during heating from 130 to 280 °C for neat rPP and rPP-MCF composites; low MCF loading strongly lowers the viscosity in the processing-relevant range, while higher loadings partially rebuild the viscosity but keep it below the reference rPP.

In the MCF-filled series (Figure 15), the viscosity–temperature curves of all formulations remain below that of the reference rPP over the whole processing window. At 230 °C, rPP MCF2 exhibits a strongly reduced viscosity of 66 Pa·s, in line with the observed increase in MVR/MFR and indicating that a small amount of short microcellulose fibres facilitates chain mobility and effectively acts as an internal processing aid [23]. Increasing the fibre content to 5 and 10 pbw leads to viscosities of 59 and 355 Pa·s, respectively, which reflects the gradual formation of a sparse fibrous network that adds hydrodynamic resistance to flow while still keeping the overall processability close to that of the neat matrix [23].

**Figure 16:**
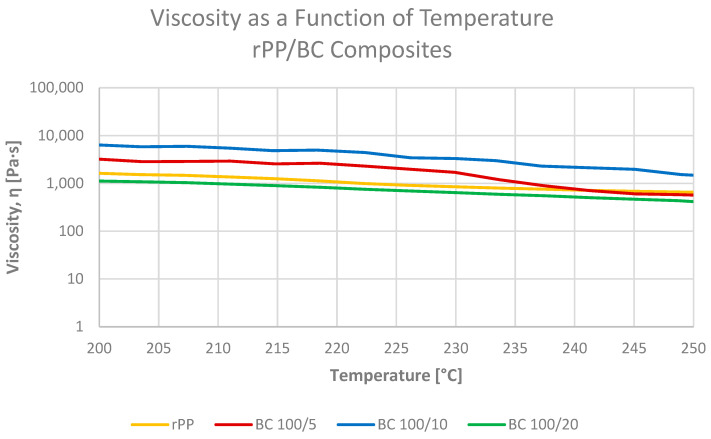
Apparent melt viscosity as a function of temperature during heating from 130 to 280 °C for neat rPP and rPP-BC composites; 5 and 10 pbw BC markedly increase viscosity around 230 °C, whereas 20 pbw BC shows a reproducible decrease, attributed to agglomeration and cluster slip at high filler concentration.

The effect of microcellulose-derived biochar is stronger and more complex (Figure 16). At 230 °C, the viscosity of rPP BC5 and rPP BC10 rises to 1408 and 3394 Pa·s, respectively, which confirms a pronounced stiffening of the melt with increasing BC content [12,43]. In contrast, the highest loading, rPP BC20, shows a lower apparent viscosity of 609 Pa·s, i.e., below the neat rPP value, and this behaviour was reproduced in several independent runs, indicating that it is a systematic feature of the material rather than an experimental outlier. Similar non-monotonic trends have been reported for highly filled polymer systems, where, above a critical filler concentration, the primary particle network gives way to larger, loosely packed agglomerates that can slide against each other under low shear [44,45]. In such a state, the rheometer mainly records the motion of these clusters rather than a homogeneous, strongly connected network, which leads to a reduced apparent viscosity, even though the solid fraction is higher [45].

This interpretation is consistent with the MFI results discussed in Section 3.3.1. MFI measurements at 230 °C and 2.16 kg show a strictly monotonic decrease in MVR/MFR from rPP BC5 through BC10 to BC20, demonstrating that under high, confined shear, the composite with 20 pbw BC offers the largest overall flow resistance. In contrast, the temperature-dependent melt viscosity experiment probes the melts at a much lower shear rate and in a large-gap geometry, where the highly filled BC20 composition can reorganize into agglomerated, cluster-like domains and exhibit local slip instead of coherent flow [44,45]. Consequently, the MFI and ARES data should be regarded as complementary: MFI reflects the behaviour under industrial capillary processing, whereas ARES reveals how the structure of the BC network evolves at near-rest conditions and highlights the onset of agglomeration-dominated flow at the highest biochar loading.

### 3.4. Water Contact Angle

**Figure 17:**
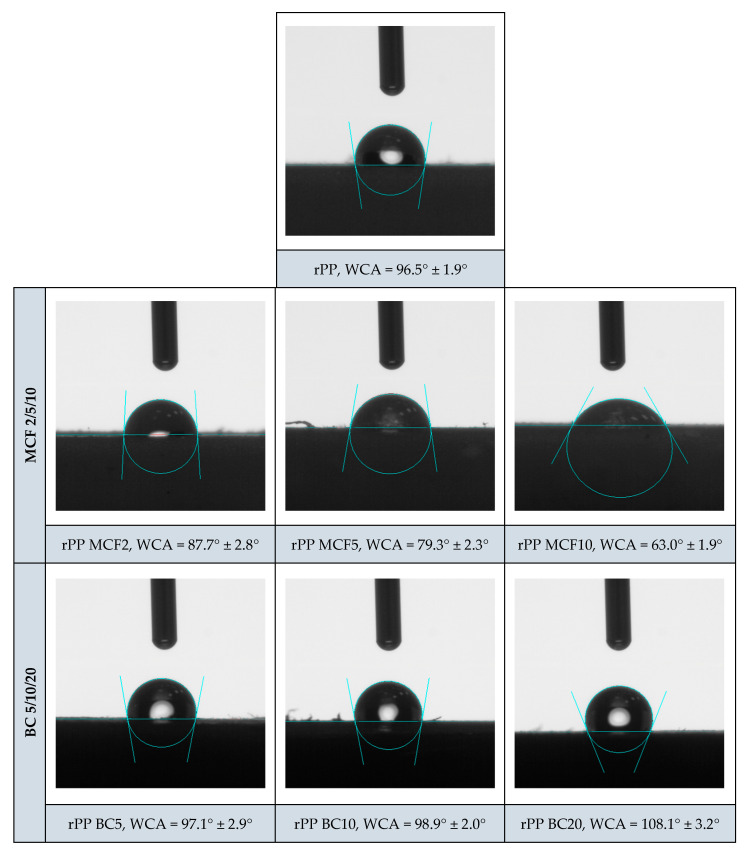
Static water contact angle (WCA) images of neat recycled polypropylene and rPP composites filled with microcellulose fibres and microcellulose-derived biochar. MCF progressively increases surface hydrophilicity with increasing fibre content, whereas BC systematically raises surface hydrophobicity above the neat rPP level.

Static water contact angle measurements were used to assess how the incorporation of microcellulose fibres (MCFs) and microcellulose-derived biochar (BC) modifies the surface wettability of the recycled polypropylene matrix (Figure 17). Neat rPP exhibits a relatively high average water contact angle of 96.5° ± 1.9°, indicating a hydrophobic surface typical of non-polar polyolefins [46,47].

The introduction of MCF leads to a pronounced, systematic reduction in contact angle with increasing fibre loading: from 87.7° ± 2.8° for rPP MCF2 to 79.3° ± 2.3° for rPP MCF5 and down to 63.0° ± 1.9° for rPP MCF10. This decrease can be attributed to the increasing exposure of cellulose-rich domains at or near the composite surface, which promotes polar interactions with water and consequently enhances surface wettability [48,49]. Such behaviour is consistent with previous reports on cellulose systems, where higher fibre contents generally translate into lower water contact angles due to the progressive enrichment of hydrophilic domains at the polymer–air interface [50,51,52].

In contrast, the BC-filled composites retain or even slightly exceed the hydrophobicity of neat rPP. For rPP BC5 and rPP BC10, the average water contact angles remain close to or slightly above that of the matrix, at 97.1° ± 2.9° and 98.9° ± 2.0°, respectively, while rPP BC20 reaches 108.1° ± 3.2°. This trend indicates that the carbonised, largely non-polar biochar surface does not introduce additional hydrophilic functionality; instead, at higher loading, it can slightly increase the apparent hydrophobicity of the composite surface [12,53]. The increase in WCA observed at 20 pbw BC indicates that the composite surface becomes increasingly dominated by biochar-rich regions and/or a roughened carbonaceous morphology, which limits water spreading. This behaviour is consistent with literature reports describing biochar as a relatively hydrophobic, carbon-rich filler in polyolefin matrices.

### 3.5. Microstructure Characterization by Optical Microscopy

**Figure 18:**
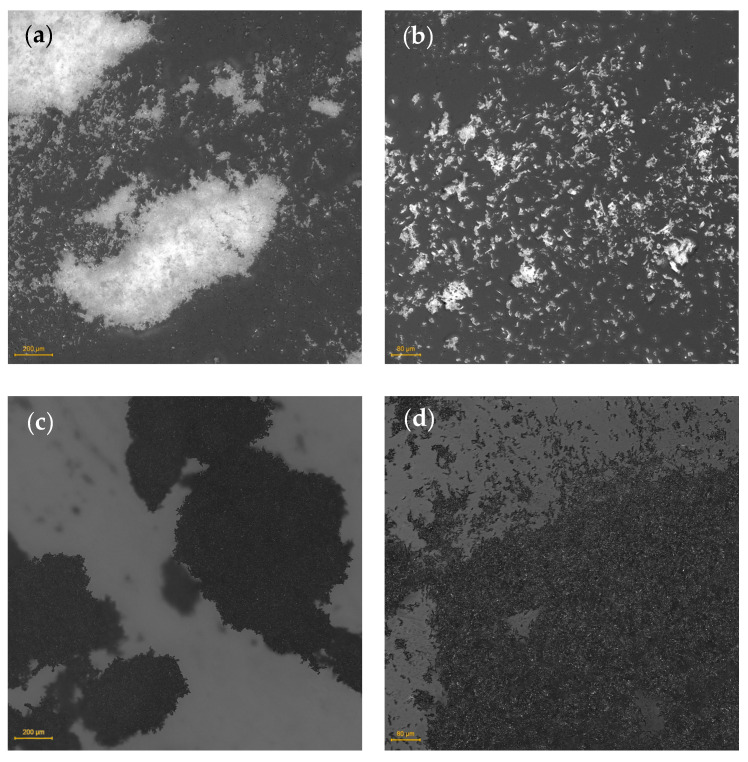
Optical micrographs of the neat biofillers at two magnifications: (**a**) MCF, 50×; (**b**) MCF, 100×; (**c**) BC, 50×; (**d**) BC, 100×. Elongated, fibre-derived fragments are visible in both materials, indicating that pyrolysis largely preserves the parent MCF morphology whilst converting the fibrous cellulose structure into a compact, carbonaceous phase.

The optical micrographs in Figure 18 present the standalone bio-fillers: microcellulose fibres (MCFs, 6–12 µm) and the corresponding biochar (BC) obtained by pyrolysis. At 50× magnification, both materials form irregular agglomerates, but the MCF regions appear more diffuse, whereas BC domains are more compact, indicating a higher packing density of the carbonised phase.

At 100× magnification, MCF reveals distinctly elongated, fibre-like fragments with well-defined boundaries, consistent with the high-aspect-ratio morphology of the Arbocel UFC 100 grade. Analogous elongated structures remain clearly discernible in the BC micrographs, albeit with markedly darker contrast, indicating that pyrolysis at 600 °C largely preserves the fibre-derived morphology at the microscale. This observation aligns with previous reports demonstrating that cellulose-derived biochar produced within the 500–700 °C range retains the fibrous architecture of the precursor material [27].

The morphological continuity between MCF and BC has direct implications for the composite properties reported in the preceding sections. The preserved anisotropic particle geometry of BC may contribute to load transfer within the rPP matrix in a manner analogous to short-fibre reinforcement, providing a structural basis for the comparable trends in flexural modulus and impact strength observed for both filler series [54]. Simultaneously, the removal of oxygen-containing surface groups during carbonisation renders BC hydrophobic, which accounts for its opposite effect on water contact angle relative to MCF, as discussed in Section 3.4 [54,55].

## 4. Conclusions

This work demonstrated that recycled polypropylene from post-industrial BOPP packaging films can be effectively upgraded using bio-based fillers derived from a single cellulose precursor, namely microcellulose fibres (MCFs) and microcellulose-derived biochar (BC). Both fillers acted as efficient heterogeneous nucleating agents, slightly increasing the crystalline fraction and shifting crystallization to higher temperatures without significantly changing the melting behaviour of rPP, which is beneficial for dimensional stability during processing.

MCFs and BC provided distinct yet complementary property profiles. MCFs introduced an additional low-temperature degradation step and lowered T_5_, but at moderate loadings increased MVR/MFR and reduced melt viscosity, thereby facilitating flow while simultaneously increasing tensile and flexural moduli and markedly improving impact strength at 10 pbw. In contrast, BC increased T_5_ and DTG peak temperatures, generated a substantial char residue at 20 pbw, and strongly stiffened both the melt and solid state, delivering higher modulus gains (≈40–50%) at the expense of ductility at the highest contents.

Water contact angle measurements confirmed that the two fillers also tune surface properties in opposite directions: MCF significantly increased surface hydrophilicity, whereas BC maintained or slightly enhanced hydrophobicity. Overall, converting one cellulosic feedstock into either fibrous or carbonised fillers offers a versatile toolbox for tailoring the crystallization behaviour, thermal stability, rheology, mechanical performance and surface wettability of rPP, opening composition windows in which upgraded recyclate can re-enter more demanding applications, in line with circular economy principles.

## Figures and Tables

**Table 1:** Formulations of recycled polypropylene (rPP) composites containing microcellulose fibres (MCFs) or biochar (BC), expressed per 100 parts by weight (pbw) of rPP.

Sample	rPP [pbw]	Micro-Cellulose Fibres (MCF) [pbw]	Biochar (BC) [pbw]	Bio-Filler [wt %]
rPP	100	-	-	0
rPP MCF2	100	2	-	2.0
rPP MCF5	100	5	-	4.8
rPP MCF10	100	10	-	9.1
rPP BC5	100	-	5	4.8
rPP BC10	100	-	10	9.1
rPP BC20	100	-	20	16.7

**Table 2:** Crystallization temperatures and crystallinity degree Xc of recycled polypropylene and rPP composites with microcellulose fibres (MCFs) and microcellulose-derived biochar (BC) obtained from DSC cooling scans.

Sample		Tc_onset_ [°C]	Tc_peak_ [°C]	Tc_endset_ [°C]	ΔHc [J/g]	Xc [%]
rPP		120	115	108	88.6	42.8
rPP MCF	100/2	122	118	111	84.9	41.5
	100/5	126	121	113	80.6	40.6
	100/10	127	122	116	76.3	40.5
rPP BC	100/5	125	121	114	82.6	41.7
	100/10	126	122	117	78.9	40.9
	100/20	128	123	117	73.0	42.3

Tc_onset_—the onset temperature of crystallization, Tc_peak_—temperature of the peak of crystallization, and ΔHc—enthalpy of the crystallization process.

**Table 3:** Melting temperatures and crystallinity degree Xc of recycled polypropylene and rPP composites with microcellulose fibres (MCFs) and microcellulose-derived biochar (BC) obtained from the second DSC heating scans.

Sample		Tm_onset_ [°C]	Tm_peak_ [°C]	Tm_endset_ [°C]	−ΔHm [J/g]	Xc [%]
rPP		153	165	173	63.2	30.6
rPP MCF	100/2	153	165	172	65.1	32.0
	100/5	153	165	171	72.9	37.0
	100/10	154	166	171	67.7	35.8
rPP BC	100/5	154	165	171	64.1	32.5
	100/10	155	163	170	64.7	33.4
	100/20	154	165	171	61.7	35.8

Tm_onset_—the onset temperature of melting, Tm_peak_—temperature of the peak of melting, and −ΔHm—enthalpy of the melting process.

**Table 4:** Thermogravimetric parameters of neat rPP, microcellulose (MCF) and rPP composites with MCF and microcellulose-derived biochar (BC): temperature at 5% mass loss (T_5_), temperature at the DTG maximum (T_DTG_), and total mass loss in the inert stage between 25 and 600 °C (ΔM_25–600_).

Sample		T_5_ [°C]	T_DTG_ [°C]	ΔM_25–600_ [%]
rPP		425	469	99.9
MCF		288	352	92.7
rPP MCF	100/2	425	471	99.8
	100/5	375	471	99.7
	100/10	347	471	99.6
rPP BC	100/5	441	475	96.0
	100/10	443	477	92.4
	100/20	449	477	87.2

T_5_—the onset temperature of thermal decomposition; T_DTG_—derivative thermogravimetric peak temperature.

## Data Availability

The original contributions presented in the study are included in the article, further inquiries can be directed to the corresponding author.
